# Chemical grafting of waste polystyrene with acrylic acid and subsequent amine functionalization to enhance polystyrene modified bitumen characteristics

**DOI:** 10.1038/s41598-026-57285-9

**Published:** 2026-06-17

**Authors:** Z. L. Abo-Shanab, Elsayed A. Elsharaky, Amira E. El-Tabey, A. A. Ragab

**Affiliations:** Petroleum Application Department, Egyptian Petroleum Research Institute, Cairo, 11727 Egypt

**Keywords:** Chemical grafting, Waste polystyrene, Bitumen, Rutting factor, Storage stability, Chemistry, Engineering, Materials science

## Abstract

The rigid polystyrene (PS) waste could be an alternative bitumen modifier, which brings great economic and environmental efficiency. However, because of low polarity and high rigidity nature of PS, it sometimes poses some difficulties in compatibility, elasticity, and storage stability. To address these limitations, the present study proposed an alternative chemical modification method for waste polystyrene through grafting with acrylic acid followed by a reaction with diethylenetriamine to produce PS-g-AM. Functionalized groups of the polymer were confirmed by performing FT-IR and 1 H-NMR spectroscopy which approved that PS-g-AM was successfully synthesized and identified. The synthesized PS − g−AM was mixed with bitumen with percentages of 3, 5, 7 and 10wt% from total binder weight. A multi-scale correlation between microscopic mechanisms and macroscopic performance is integrated. The microscopic characterization including FTIR and AFM techniques were performed and successfully verified the good compatibility via hydrogen bonding interaction between PS-g-AM and bitumen. Also, the macroscopic characterization including storage stability, and elastic recovery test demonstrated that the prepared PS-g-AM modified binders (7wt%) showed significantly a high storage stability (≤ 0.3), and better elastic recovery (55%) compared to untreated PS modifier (2.5), and (25%) respectively. The dynamic mechanical properties are also studied and the results revealed a considerable improvement in rutting resistance (G/sin δ) PG degree increased from 70 to 76, tan δ reduction. In summary, results confirmed that chemical grafting turned PS from a hardening agent to an efficient elastic modifier into asphalt and provided great opportunity for a high-performance sustainable method of modifying the asphalt.

## Introduction

Conventional bitumen is highly temperature-sensitive; high service temperatures lead to a viscous behavior, whereas low temperatures result in brittleness. To overcome these limitations, extensive studies have been conducted to obtain a polymer with better elastic and heat stability properties along with higher resistance to permanent deformation through chemical modification of polymers. However, published Polymer modifiers are still a relatively difficult option due to problems with compatibility, storage stability, cost and sustainability^1^. Due to the demand for sustainable pavement materials^2^ and increasing concerns about the environment, more attention has been paid to use waste plastics as asphalt modifiers in last year’s^3–8^.

Polystyrene (PS) is one of these wastes that has a low density and is poorly biodegradable, which generates huge amounts from packaging and insulating applications and raises great disposal concerns^9–12^. It has been reported in the literature that PS foam can improve bitumen’s stiffness and high-temperature performance, despite its rigid aromatic structures, non-polar characteristics, and poor elastic recovery which is often caused when PS foams are added to bitumen due to compatibility and storage stability issues at either high modifier loads or prolonged storage conditions. Unmodified PS foam well therefore usually behaves more like a stiffening agent than a useful elastic modifier^11^. Capable of solving these issues, recent studies have focused on the importance of the chemical functionalization of polymers for better interactions with bitumen constituents^13,14^. The presence of polar functional groups in the polymer chains will significantly enhance their compatibilization with bitumen, in particular its polar fractions (resins and asphaltenes) due to hydrogen bonding and dipole-dipole interactions^15^. This technique further increases dispersion and storage stability, as well as the elastic behavior by facilitating better stress transfer between polymer and binder matrix^15,16^. Chemical grafting rather than physical blending can provide improved rheological performance and permanent homogeneity. As amines are grafted onto the polymer backbones, they create amphiphilic moieties to convert stiff thermoplastics into more elastic materials with enhanced interfacial adhesion. However, limited studies have been conducted on the chemical grafting of waste polystyrene with amine derivatives to modify asphalt binder. Most new studies are based on unmodified PS or for classical elastomers such as styrene butadiene styrene rubber (SBS)^17^ which, although useful, present challenges through their high costs and storage stability problems^16,18^. It is important to keep that although the polystyrene backbones of virgin and waste polystyrene are equal, they are not inherently more compatible with bitumen. The amphiphilic grafting technique, which may be used with either virgin or waste feedstock, is responsible for the enhanced compatibility seen in this study. Despite being cheap and plentiful, scrap polystyrene foam’s poor compatibility limits its immediate application. By adding polar groups that can form hydrogen bonds with resins and asphaltenes to create reversible physical crosslinks, the grafting technique significantly improves performance at a somewhat higher cost. This improves elastic recovery and stress transfer without forming a covalent link with bitumen. Therefore, waste polystyrene offers a more sustainable solution for large-scale applications by offering economic and environmental benefits without compromising performance, even though it is not intrinsically more suitable.

In this context, the present study offers a new sustainable modification approach where waste polystyrene is chemically grafted with acrylic acid, which is then treated with diethylenetriamine to yield an asphalt binder modified polymer (PS-g-AM). Instead, it proposes a two-step synthesis consisting in the formation of polystyrene-grafted acrylic acid (PS-g-AA) by grafting polystyrene with acrylic acid and molecular conversion of carboxylic groups into amide functionalities using diethylenetriamine to achieve this synergetic effect between the inherent stiffness contribution brought by the polystyrene backbone and better polarity and elasticity provided by each amide group. The two-step method (grafting then amidation) allows independent control of grafting degree and amide functionality, which is not possible in one-step reactions.

The properties of PS-g-AM-modified bitumen are systematically evaluated and compared with pure unmodified bitumen and polystyrene modified binders. Comprehensive characterization is conducted using Fourier transform infrared spectroscopy (FT-IR) and nuclear magnetic resonance (1 H-NMR) to confirm chemical grafting. Chemical compatibility with bitumen is also verified by FTIR and AFM. Conventional physical properties, penetration index, elastic recovery, and storage stability are investigated to evaluate binder homogeneity and temperature susceptibility. In addition, dynamic viscosity measurements and dynamic shear rheometer (DSR) tests are performed to assess rheological behavior and rutting resistance before and after short-term aging.

## Materials and experimental methods

### Materials

Local bitumen binder (blank) of penetration grade (AC 60/70) produced by El-Nasr Petroleum Company in Suez, Egypt. Polystyrene trash was derived from food packaging waste (the waste was collected and wash to remove any food residual). Sigma-Aldrich Company contributed acrylic acid, diethylene triamine, and benzoyl peroxide (BPO).

### Experimental methods

#### Synthesis and characterization of PS-g-AM

##### Grafting of waste polystyrene

10 gm of waste polystyrene were dissolved in a suitable amount of xylene and added to the flask. The flask was purged with dry nitrogen gas constantly. 0.1 g of benzoyl peroxide, chosen as the initiator, were added to the flask. Next, 7 gm of acrylic acid monomer was added to the reaction solution. The reaction solution was stirred at a temperature of 90 °C for duration of 2 h. To terminate the reaction, ethanol was added to the flask. The resulting mixture was then filtered to separate the grafted waste polymer, known as PS-g-AA. It appeared as a white solid. The PS-g-AA product was thoroughly washed with water and ethanol to remove any residual impurities. Finally, the grafted waste polymer was dried under vacuum conditions at a temperature of 70 °C (yield = 60%).

##### Amidation of PS-g-AA with diethylene triamine

Diethylene triamine (10 gm) was introduced drop by drop to 7 gm of grafted polymer which dissolved in xylene in presences of boric acid as catalyst. The reaction mixture was agitated for two hours at 140 °C. The resultant product was washed several times with water, ethanol and acetone to give finally polymer which named PS-g-AM (yield = 82).

##### Confirmation the chemical structure

FTIR and ^1^H-NMR spectroscopy were employed to validate the chemical structures of the synthesized polymers. FTIR spectra were recorded on a Thermo Scientific Nicolet iS-10 spectrometer using the KBr pellet method. ¹H-NMR spectra were obtained at 400 MHz on a Bruker Avance (III) NMR spectrometer, utilizing deuterated dimethyl sulfoxide (DMSO-d₆) as the solvent.

#### Modification of Bitumen with PS-g-AM

In this step, the calculated amount of bitumen was heated to 180 °C in a small container until it softens and become pourable. The calculated amounts of PS-g-AM depending on the addition percent as 3wt%, 5wt%, 7wt% and 10wt% (from the total weight of the blank bitumen) were gradually added to molten bitumen in a constant rate as 5 g/ min under high shear mixer rotating at 4000 rpm for 0.5 hrs at controlled temperature 165 °C until the blends became essentially homogenous. After mixing, the modified binder was poured into silicone molds and cooled at room temperature for 0.5 hour, then stored in sealed containers at ambient temperature (25°C) for 24 h prior to testing.”

#### Physical properties of bitumen modified by PS-g-AM

The physical properties were studied including penetration at 25 °C according to ASTM D 5, softening point test according to ASTM D 36, and kinematic viscosity as recommended by ASTM D2170. The dynamic viscosities were also investigated via a stress-controlled rheometer (Rheometer, Modulated Compacted Rheometer (MCR 52), Anton Paar Instruments, Austria) according to ASTM D4287. Storage stability according to ASTM D7173. the samples are then subjected to short term aging process using RTFO according to ASTM D2872. Asphalt sample is placed on thin film oven which was equipped with a thermostat and a rotating disc rack at 163 °C for 5 h.

#### .AFM topography

Two samples were prepared from Bitu+7wt%PS and Bitu+7wt% PS-g-AM. They were dissolved in 5wt% toluene, spun onto mica substrates for 60 s at 2000 rpm, and vacuum-dried for 24 h at 40 °C. Using a [Flex AFM] device with a scan size of [2.5] µm × [2.5] µm and a scan rate of 0.8 Hz, AFM imaging was carried out in tapping mode. Ra, Rq, and Rmax values were determined by flattening and analyzing height pictures. To guarantee reproducibility, three regions were scanned for every sample.

#### Dynamic shear rheometer

Triton Technology-TTDMA was used to conduct the dynamic shear rheometer test in accordance with ASTM D-7175 requirements. After two hours of heating at 135 °C, all bitumen samples are put into silicon rubber molds to create samples with an 8 mm diameter and a 2 mm thickness. The samples are then left to cool for around an hour. provides information on sample preparation and testing for dynamic shear analysis^19,20^. To reduce any historical burden related to sample preparation, loading, and handling, samples were pre-shared and loaded at a temperature six degrees Celsius below the testing temperature. At a fixed frequency of 10 rad/sec, temperature sweeps ranging from 40 °C to 90 °C with 5 °C increments were applied. The factor G*/sin δ, where G* is the complex shear modulus and δ is the phase angle, was used to quantify the asphalt binder’s resistance to permanent deformation at high temperatures (rutting resistance). The test is performed on unaged asphalt binders first, followed by an RTFO test on aged binders.

## Results and discussion

### Affirmation of PS-g-AM

#### FTIR spectra

**Scheme 1:**
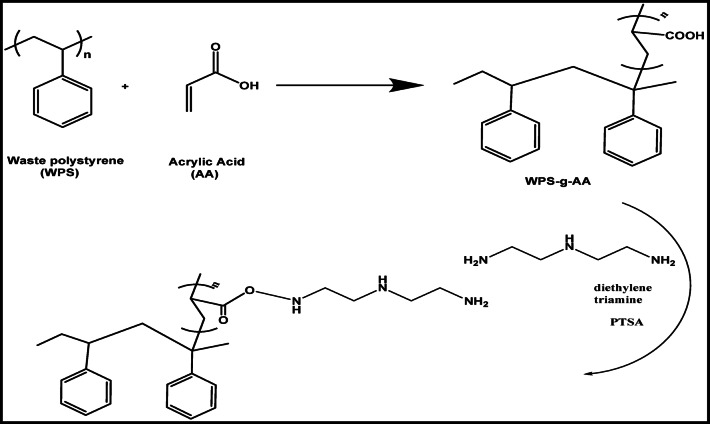
Scheme of preparation of Amphiphilic polystyrene waste.

**Fig. 1:**
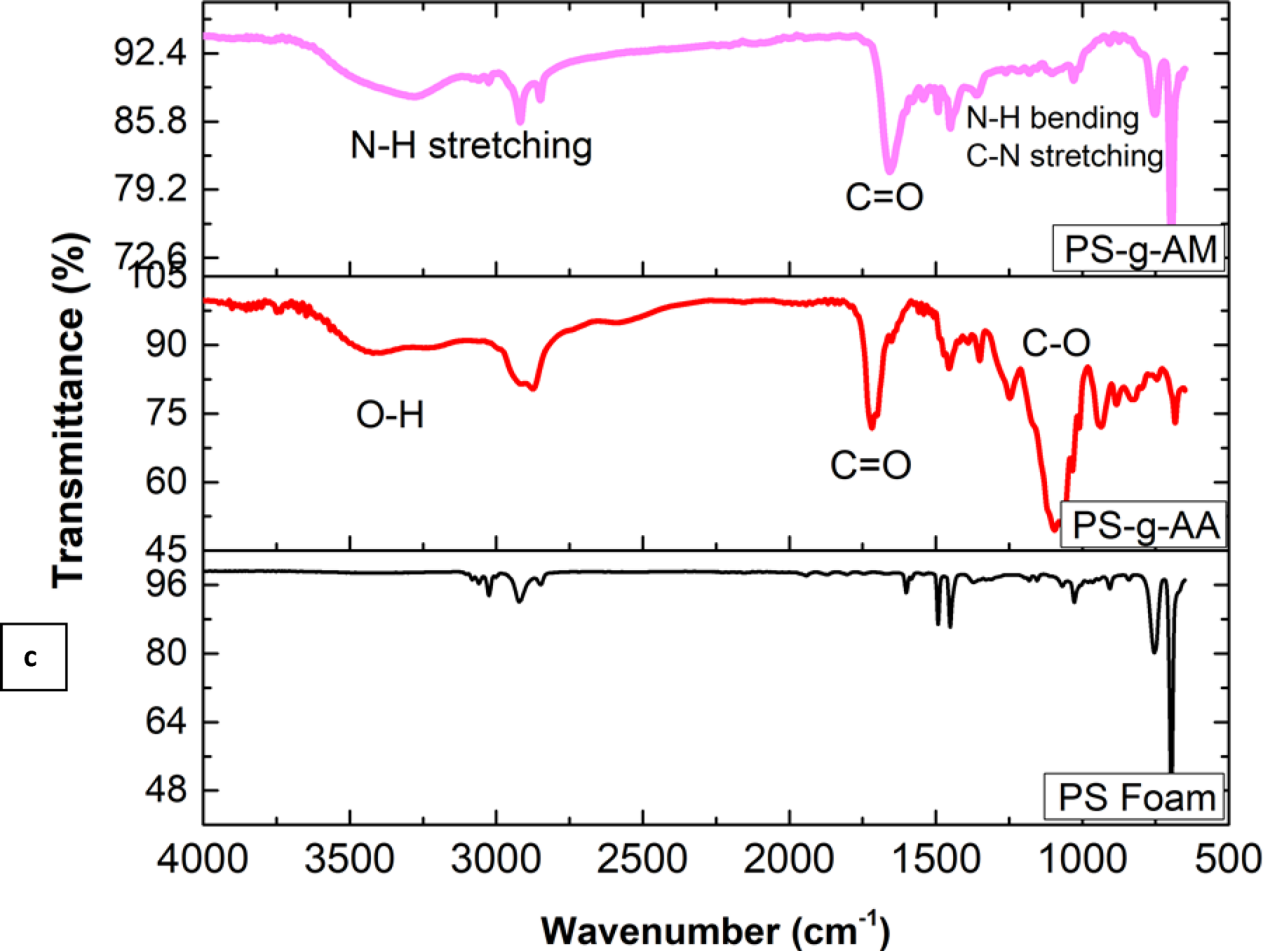
FTIR spectrum of (**a**) PS, (**b**) PS-g-AA, (**c**) PS-g-AM.

The assignment of all possible peaks is listed in Table 1. The FT-IR spectra of waste polystyrene (PS); PS-g-AA and PS-g-AM were illustrated in Figure 1 exhibited characteristic absorption bands at 3024 cm⁻¹ corresponding to aromatic C–H stretching, 2921–2849 cm⁻¹ assigned to aliphatic C–H stretching, and characteristic peaks at 1600, 1492, and 1451 cm⁻¹ attributed to aromatic C = C stretching vibrations, confirming the polystyrene backbone structure. There were no hydroxyl or carbonyl bands observable. After grafting with acrylic acid (PS-g-AA), a new strong absorption band appeared at approximately 1718 cm⁻¹ corresponding to the C = O stretching vibration of carboxylic acid groups, a broad O–H stretching band in the range of 3420 cm⁻¹ and C–O stretching bands around 1247 cm⁻¹, confirming successful grafting of acrylic acid onto the PS backbone. Following amidation with diethylenetriamine to form PS-g-AM, the carbonyl peak shifted to lower wavenumbers 1656 cm⁻¹, characteristic of amide carbonyl group (stretching of CONH), and a new band appeared at approximately 1543 cm⁻¹ corresponding to (N–H bending and C–N stretching), together with N–H stretching bands around 3279 cm⁻¹. All of these indicates that PS-g-AM was successfully synthesized, the mechanism of reaction is illustrated in Scheme 1.

**Table 1:** FTIR peak assignment of PS, PS-g-AA, PS-g-AM.

Wavenumber (cm⁻¹)	Assignment	Waste PS (a)	PS-g-AA (b)	PS-g-AM (c)	Interpretation
3020–3080	Aromatic C–H stretching	✓	✓	✓	Characteristic of polystyrene backbone
2920–2850	Aliphatic C–H stretching	✓	✓	✓	Alkyl chains in polymer structure
1600	Aromatic C = C stretching	✓	✓	✓	Benzene ring vibration (PS structure retained)
1490–1450	Aromatic skeletal vibration	✓	✓	✓	Confirms polystyrene framework
1718	C = O stretching (carboxylic acid)	-	✓	-	Strong evidence of acrylic acid grafting
3420 (broad)	O–H stretching	-	✓	-	Hydrogen-bonded carboxylic acid groups
1247	C–O stretching	-	✓	✓	Confirms presence of –C-O bond
1656	C = O stretching for amide	-	-	✓	Amide group
3279	N-H stretching	-	-	✓	Free terminal amine
1543	N–H bending	-	-	✓	Confirms amide functionality

#### ^1^H-NMR Spectra

**Fig. 2:**
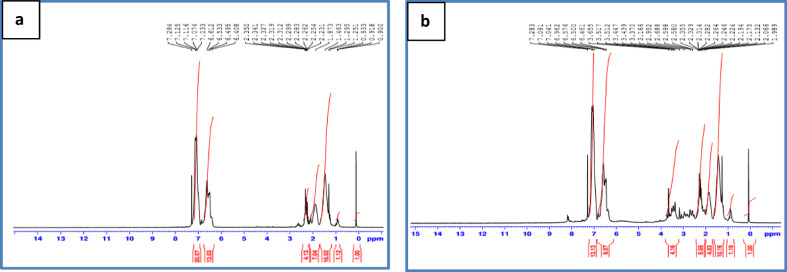
The ^1^H-NMR spectrum for (**a**) PS-g-AA, (**b**) PS-g-AM.

The ¹H NMR spectrum of grafted styrene-acrylic (PS-g-AA) was shown in Fig. 2a,b. It shows Chemical Shift (δ) at 6–7 ppm for aromatic benzene ring of styrene back bone polymer; 2.8 and 1.8 ppm for methylene protons (–C**H**–C**H**_**2**_–) of styrene part; 1.46 and 2.3 ppm for C**H**_2_-C**H**_2_COOH; and also signal from some impurities at about 0.9 ppm. The ¹H NMR spectrum PS-g-AM **Figure (2b)** shows anew signals in addition to previous signals of PS-g-AA as δ at 8.1; 3.5 and 2.6 ppm for -N**H**C**H**_2_-C**H**_2_- respectively. The FT-IR and ^1^H-NMR data were confirmed the functionalization of WPS to become amphiphilic polymer.

### Interaction between bitumen and PS-g-AM

**Fig. 3:**
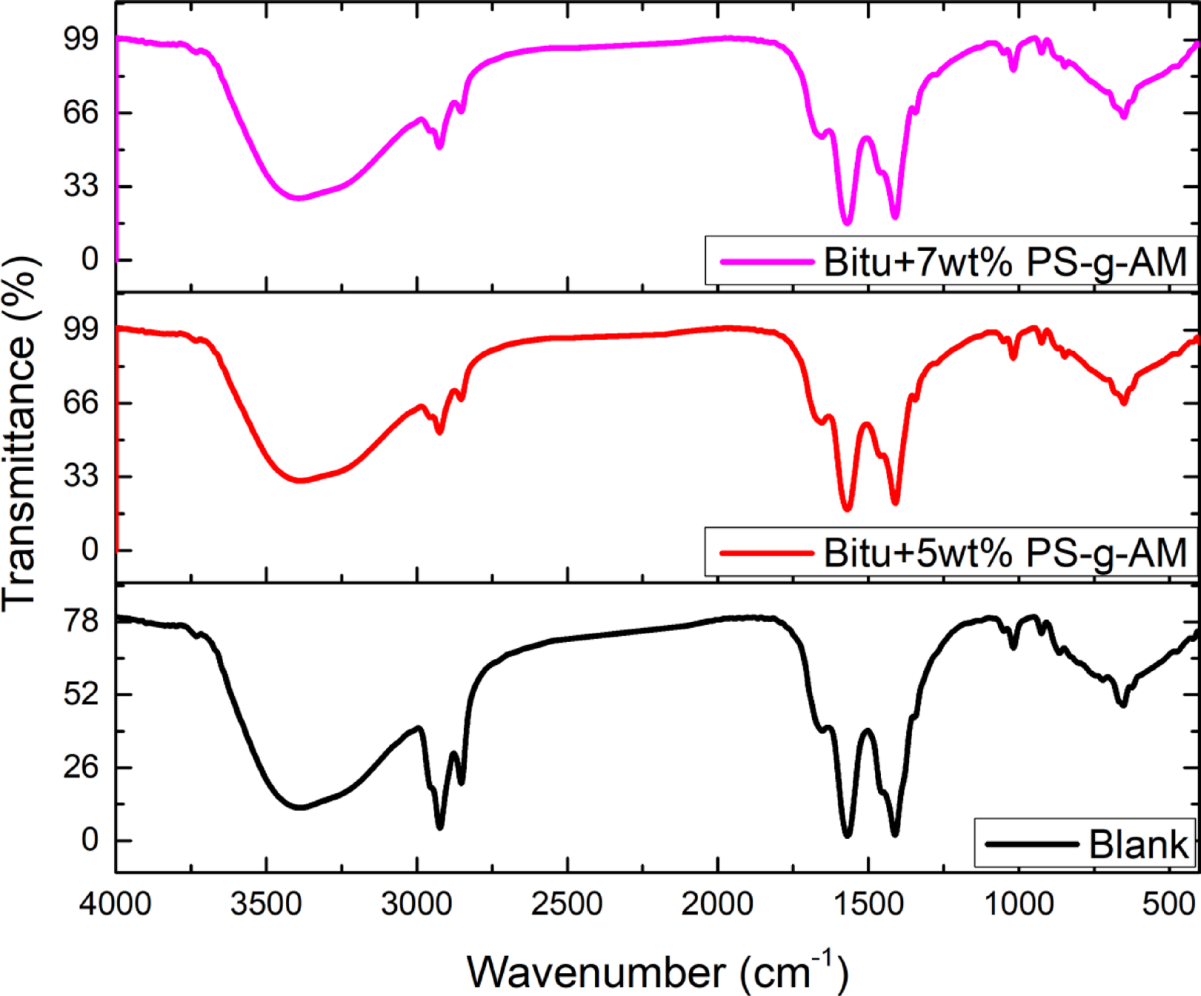
FTIR spectrum of (**a**) blank bitumen, (**b**) Bitu+5wt%PS-g-AM, (**c**) Bitu+7wt%PS-g-AM.

To determine the type of interaction that could causes a significant change in rheological properties, the chemical structures of blank bitumen, PS-g-AM, and bitumen modified by PS-g-AM were analyzed using FTIR spectroscopy as shown in Figure 3.

The presence of several absorption bands associated with bitumen’s hydrocarbon base is a characteristic of virgin bitumen’s FTIR spectra. These intricate band characteristics are a reflection of bitumen’s complex chemical makeup, which is primarily responsible for its viscoelastic behavior and is composed of saturate fractions, aromatic fractions, resin fractions, and asphaltene fractions (SARA fractions). The presence of polar functional groups that can interact strongly with the polar fractions in bitumen is confirmed by the PS-g-AM spectrum’s specific absorption bands, which are previously detailed in the affirmation section of the PS-g-AM spectrum. The amide-related absorption bands become conspicuous in the modified binders, especially in the N–H bond expansion areas (≈ 3300 cm⁻¹) and the amide I and II bands (≈ 1650 and 1550 cm⁻¹), according to the FTIR spectra of bitumen modified by 5wt% and 7wt% of PS-g-AM. These bands in modified binders seem broader and somewhat shifted when compared to the spectrum of pure PS-g-AM. This suggests that the polymer was successfully incorporated into the bitumen matrix through strong physicochemical interactions rather than straightforward physical mixing. Hydrogen bonding between amide functional groups and polar fractions in bitumen, such as resins and asphaltenes, is usually responsible for these shifts^21,22^. No new covalent bonds are created throughout the alteration process, as seen by the changed binders’ lack of new bands. Rather, the reaction mechanism is dominated by non-covalent interactions, especially polar and hydrogen bonds, which are adequate to change the binder’s microstructure and viscoelastic properties^23,24^.

### AFM topography explanation

**Fig. 4:**
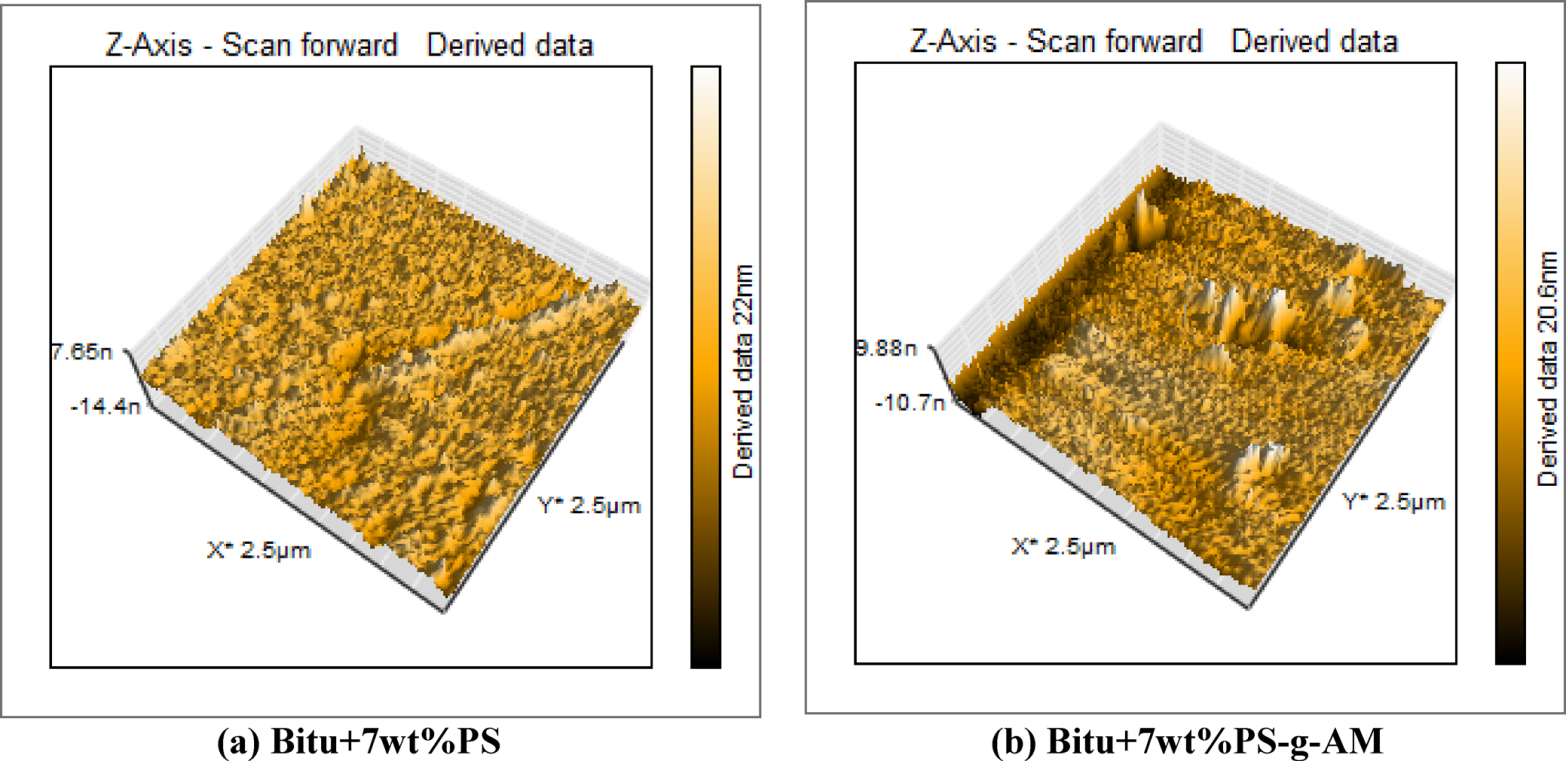
AFM of (**a**) Bitu+7wt% waste PS, (**b**) Bitu+7wt%PS-g-AM.

The topography of bitumen loaded with 7wt% untreated polystyrene and 7wt% amine functionalized polystyrene graft acrylic acid (PS-g-AM) was examined using Atomic Force Microscopy (AFM) and the data was showed in Figure 4a, b; Table 2. At the nanoscale (Ra = 4.58 nm), the AFM images (Fig. 4a) show an entirely smooth surface with no discernible characteristics (peaks). The lack of topographical characteristics suggests that the bitumen matrix and untreated polystyrene are incompatible. Because of the absence of chemical interaction and immobilization within the bitumen, the polymer is probably present as a distinct phase on the bitumen’s outside surface or as sizable domains outside the scanned area, making it impossible for AFM to identify at the nanoscale. On the other hand, PS g AM modified bitumen’s AFM pictures (Fig. 4b) show a uniformly rough surface with notable topography at the nanoscale scale (Ra = 21.91 nm). The polar functional groups (introduced by acrylic acid grafting and subsequent amidation) and the polar components of bitumen (resins and asphaltenes) interact chemically to produce this noticeable roughness as in Table 2. AFM imaging at the nanoscale is made possible by the chemical bonding, which guarantees that the functionalized polymer is properly intercalated and fixed inside the bitumen matrix^25,26^.

**Table 2:** Surface roughness data for AFM image.

Parameter	Bitu+7wt% PS	Bitu+7wt% PS-g-AM
R_a_ (nm)	4.58	21.911
R_q_ (nm)	5.96	25.89
R_y_ (nm)	28.19	102.5
R_p_ (nm)	9.02	77.34
R_v_ (nm)	-19.16	-25.6
R_m_ (nm)	-19.35	-15.89

### Physical properties of bitumen modified with PS-g-AM

#### Penetration and softening point

The results in Table 3 clearly show how the physical properties of the bituminous binder are greatly affected by the addition of PS-g-AM. The results reveal a clear trend: as the PS-g-AM concentration increases from 3% to 10%, the bitumen penetration value decreases noticeably, going from 62 dm for pure bitumen to 35 dm at 10wt% of PS-g-AM. This indicates that bitumen’s stiffness has significantly increased. This finding is also supported by the softening point data. When 7% PS-g-AM is added, the softening point of basic bitumen increases from 50.6 °C to 60 °C.

The increased softening point indicates higher resistance to flow at elevated temperatures, which is desirable for rutting resistance. However, the bitumen modified by 10wt% of PS-g-AM show low workability and high stiffness that may cause cracking at low temperature so 7wt% of PS-g-AM is considered the best choice for bitumen modification.

Table 4 show a comparative analysis between physical properties of bitumen modified by 7wt% PS-g-AM and by bitumen modified by 7wt% of untreated PS compared to blank bitumen. Both of modified binders demonstrate a significant improvement in softening point from 50 °C to 60 °C in comparison to the blank binder. Nonetheless, a significant distinction in the two modifiers’ performances is made clear by the penetration test results. The bitumen modified with untreated polystyrene (PS) shows a discernible decrease in penetration with a value of 23 dmm. This significant decrease suggests a brittle, excessively stiffened matrix where the inflexible, non-polar PS particles mostly serve as inert fillers, restricting bitumen flow and producing a largely rigid, On the other hand, the penetration value of the binder modified with the functionalized polymer (PS-g-AM) is 48 dmm. A more balanced viscoelastic character appears to be imparted by the chemically grafted acrylamide functionalities, where the flexibility and improved compatibility of the polar amide and amines groups effectively temper the stiffening contribution from the polystyrene backbone. This reduces the excessive brittleness linked to the unmodified PS and produces a binder with maintained flexibility and a better balance between high-temperature stiffness and intermediate-temperature workability^27^.

**Table 3:** Physical characteristics of bitumen using PS-g- AM.

Characteristics	Blank	PS-g-AM content								
		SD	3wt%	SD	5wt%	SD	7wt%	SD	10wt%	SD
Penetration (at 25 °C, 100 g,5s)0.1 mm	62	0.326	58	0.286	52	0.355	48	0.285	35	0.124
Softening point (ring and ball), Cº	50.6	0.408	54	0.169	58	0.249	60	0.244	62	0.047
Specific gravity (at 25 Cº)	1.02	0.86	1.027	0.012	1.033	0.082	0.038	0.001	0.042	0.002
Elastic Recovery at 25 °C (%)	< 10	-	30	0.492	50	0.571	55	0.498	60	0.124
Ductility at 25 °C (cm)	> 100	-	92	0.169	86	0.077	75	0.293	55	0.121
Viscosity at 135 °C (Pa.s)	0.45	0.008	1.20	0.081	2.20	0.081	2.50	0.057	4.00	0.120
Penetration Index	-0.51	-	0.118	-	0.719	-	0.926	-	0.57	-

**Table 4:** Comparative study between (Bitu + 7 wt% waste PS) and (Bitu+7wt% PS-g-AM).

Property	Bitumen Blank	SD	Waste PS (7 wt%)	SD	PS-g-AM (7 wt%)	SD
Penetration @25°C (dmm)	62	0.326	23	0.368	48	0.285
Softening Point (°C)	50	0.408	60	0.408	60	0.244
Penetration index	-0.5	-	-0.63	-	0.926	-
Ductility @25°C (cm)	> 100	-	40	0.326	75	0.293
Dynamic Viscosity @135°C (Pa·s)	0.45	0.008	3.5	0.073	2.5	0.057
Elastic Recovery @25°C (%)	< 10	-	25	0.163299	55	0.498063
Storage Stability ΔSP (°C)	0	-	2.5	0.040825	≤ 0.3	-
Phase Compatibility	-	-	Moderate	-	High	-

#### Penetration index

The penetration index (PI) value − 0.5 indicates a typical degree for temperature susceptibility of the blank bitumen that is representative of the conventional pavement bitumen grade. Adding polystyrene (PS) results in a further decline in the PI to -0.63, suggesting that temperature susceptibility has increased even further. This deterioration is attributed to poor compatibility and phase separation between the non-polar polystyrene and the bitumen matrix, resulting in an inert filler effect rather than true modification. In contrast, the chemically grafted polystyrene modified binder (PS-g-AM) has a positive PI value of + 0.926, which represents lower susceptibility to temperature compared with unmodified binder which are highly desirable for high-temperature pavement performance. The improvement is attributed to chemical bonding between the polar functional groups (amide/amine) of PS-g-AM and the polar components (asphaltenes, resins) of bitumen, forming a stable elastic network that resists temperature-induced softening.

#### Ductility

At Table 4 the most important difference between both untreated and treated polystyrene can be found in the comparison of ductility data. 35 cm further reduces the ductility of the unmodified PS. This substantial decrease very clearly indicates an increase in material brittleness and increased vulnerability to low-temperature cracking, since the stiff polystyrene domains act as “stress concentration” points that inhibit cohesive flow and encourage brittle fracture. In contrast, the functionalized PS-g-AM binder maintains 75 cm of ductility. The grafted functional groups (amide and amine) are responsible for maintaining the cohesive elongation. Flexible, polar > > Side chains for enhanced intermolecular interaction with the polar portion of the asphalt and high compatibility in general. More importantly, they acted not only as internal plasticizers, but also as sites of tensor relaxation in the modified matrix that improve mechanical stress redistribution and molecular mobility. This prevents crack initiation and propagation in hard particle-filled systems which maintains the thermoplastic and adhesive integrity of the binder during tensile strain^28^.

#### Dynamic viscosity

The rheological fundamental difference between untreated and chemically treated PS can be illustrated by analyzing the rotational viscosity at a 135 °C. Table 3 illustrates that the high viscosity of 3.5 Pa·s for unmodified polystyrene (PS) binder indicates that flow of bitumen is highly impeded by rigid and incompatible PS domains. As can been seen, the viscoelastic (PS-g-AM) has moderate but significant viscosity of 2.5 Pa·s, this difference reminds us important trade-off on the performance, that is due to with PS that is effectively decreasing practical workability and high stiffening. The PS-g-AM modification, on the other hand, produces a more balanced rheological profile. It maintains a viscosity more suited for routine paving operations while providing the necessary high-temperature stiffness, as seen by the increased softening point and rutting resistance. Without imposing needless practical restrictions on mixture creation and placement, this improved balance offers greater resilience to persistent deformation.

**Fig. 5:**
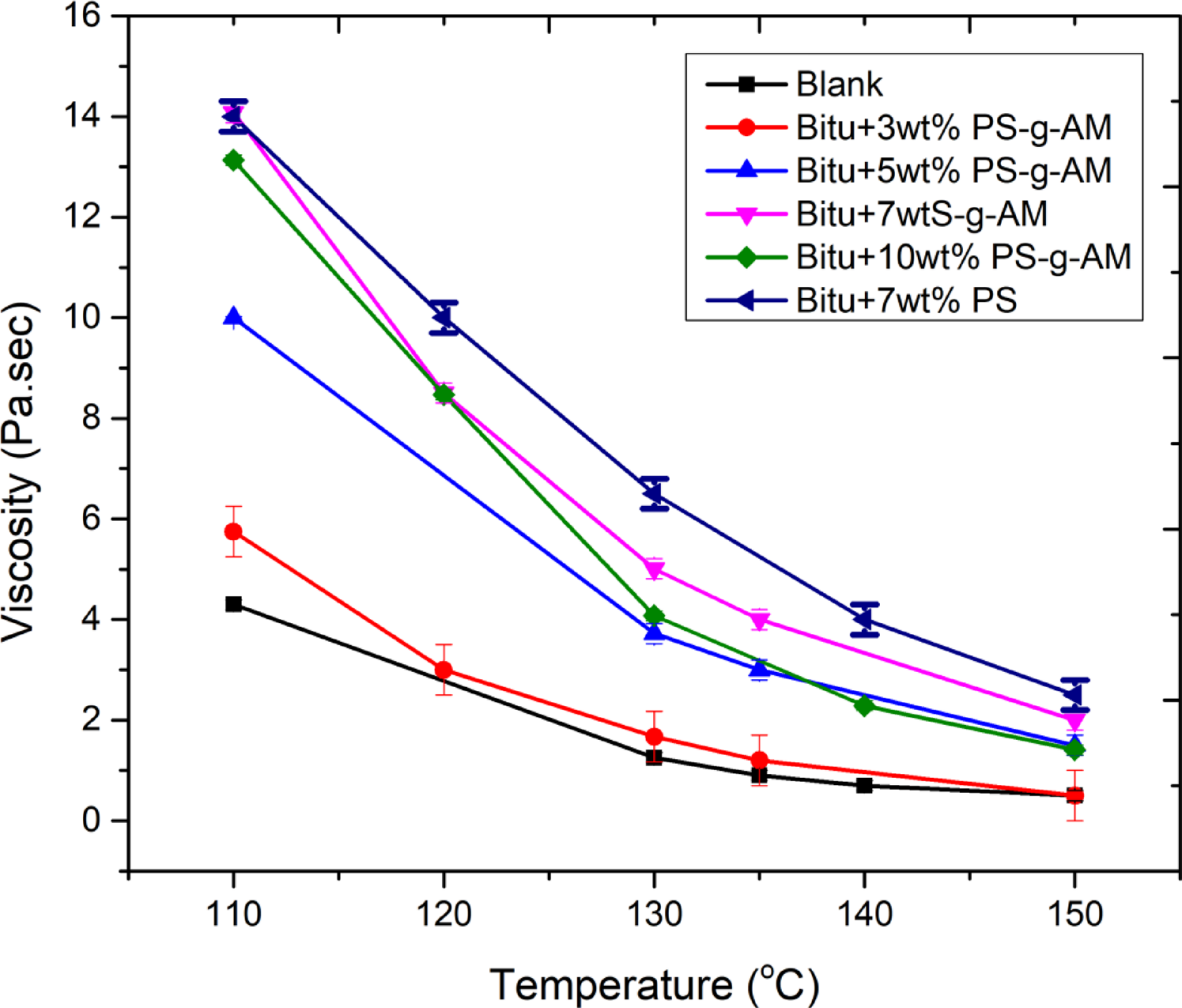
High-shear dynamic viscosity of blank, bitumen modified by PS and PS-g-AM bitumen over a temperature range of 100–150 °C.

The impact of chemically grafted acrylamide polystyrene content on bitumen’s high-shear viscosity throughout a temperature range of 100 to 150 °C is depicted in Figure 5. The bitumen was modified with PS-g-AM at contents between 3wt% and 10 wt% of the bitumen’s overall weight. In line with bitumen’s thermo-rheological behavior under high shear circumstances, it is noticed that viscosity drops for all samples as temperature rises.

Throughout the whole temperature range, the unmodified bitumen has the lowest viscosity, suggesting that it has little resistance to deformation at high temperatures. On the other side, PS-g-AM greatly increases high-shear viscosity, indicating a successful interaction between the bitumen matrix and the modified polymer. The inclusion of amide functional groups, which encourage stronger physicochemical interactions including hydrogen bonding and polar–polar interactions with the polar components of bitumen (resins and asphaltenes), that is responsible for this enhancement.

The (bitu + 7 wt% of PS-g-AM) has the best viscosity 2500 (mPa.sec) at 135 ℃ for both mixing and rutting resistance under standard Superpave PG standards, outperforming all other modified bitumen samples. This suggests that a well-developed reinforcing network has formed inside the bitumen matrix. This demonstrates that the improved PS-g-AM is optimally distributed at this content and successfully contributes to load transfer and flow resistance under high shear. Depending on the particular PG grading and local climate circumstances, it is seen that modification by 5% and 10% PS-g-AM show lower 2200 (mPa.sec) or slightly higher viscosity 3500 (mPa.sec), respectively^29,30^.

#### Elastic recovery test

Unlike simple rigid stiffening, the elastic recovery test provides an equivocal proof of actual elastic modification mechanism. As illustrated in Table 4 the low recovery of 25% for the binder loaded with 7wt% untreated polystyrene (PS) is comparable to that of an inert particle filler. With minimal elastic energy storage, this small recovery demonstrates that viscoelastic flow and plastic rearrangement of the bitumen matrix around the hard PS inclusions are the main mechanisms that accommodate any transient deformation. By contrast, a much higher elastic recovery rate of 55% is attained by the functionalized PS-g-AM modified binder. This notable improvement shows that the material’s response to stress is significantly altered by chemical grafting of amide functions. The pliable amide side chains can extend and retract reversibly, acting as molecular springs. Additionally, their polar nature facilitates strong interfacial adhesion and secondary interactions (such hydrogen bonding) with the polar components of the asphalt, thus incorporating the network of polymers into the binder matrix. By storing and recovering a significant portion of the applied deformation energy, this synergy transforms the modified polymer from a rigid fluid into a viscoelastic solid with a prominent and durable elastic component^31^. PS-g-AM (55% recovery) exhibits significantly less elasticity than commercial SBS-modified bitumen (average elastic recovery 65–75%), but it offers a sustainability advantage by using waste plastic rather than virgin SBS^30,31^.

#### Storage stability test

The transition from the marginal compatibility of PS (ΔSP = 2.5) to the near-perfect stability of PS-g-AM (ΔSP ≤ 0.3) highlights a significant advancement over conventional elastomeric modifiers like SBS. While SBS is the industry standard for bitumen modification, it is notorious for poor storage stability due to the large difference in density and solubility parameters between the polymer and the bitumen’s maltene phase. In many commercial applications, SBS-modified binders often require continuous agitation or the addition of chemical cross-linkers (such as sulfur) to prevent gross segregation during high-temperature storage.

In contrast, the PS-g-AM system achieves more homogeneity without the need for secondary cross-linking agents. The grafted acrylamide chains facilitate a robust interfacial bridge between the polystyrene backbone and the polar fractions of the bitumen (asphaltenes), effectively “anchoring” the polymer within the matrix. This chemical compatibility results in a value significantly lower than the typical to threshold often observed in unmodified SBS blends, positioning PS-g-AM as a more storage-stable and reliable alternative for long-term field applications^32^.

### Dynamic mechanical analyzing (DMA)

A dynamic mechanical analyzer (DMA) shear mode was used to assess the high-temperature rheological behavior of blank bitumen and bitumen modified by PS-g-AM with content (3wt%, 5wt%, 7wt%, and 10 wt%) before and after short-term aging by using rolling thin film oven test (RTFOT). A thorough evaluation of stiffness, elastic contribution, and resistance to permanent deformation is provided by the complex modulus (G*), phase angle (δ), and rutting parameter (G*/sin δ) obtained from the analysis of the viscoelastic response.

#### Complex modulus (G*)

**Fig. 6:**
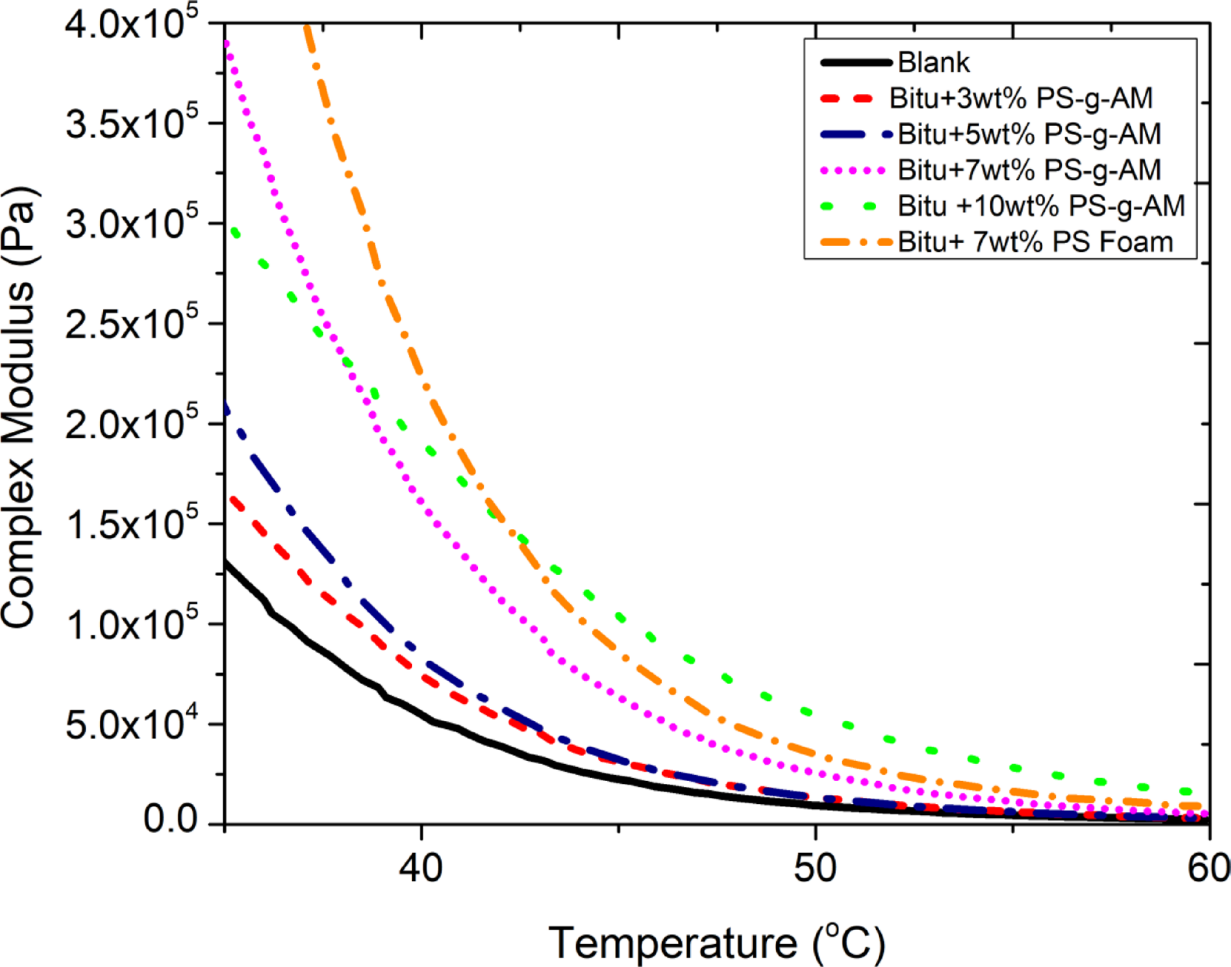
Complex modulus (G*) behavior of blank, and bitumen modified by PS, and PS-g-AM.

As shown in Figure 6 the temperature-dependent variation of the complex modulus (G*) within a temperature range of 35 °C and 60 °C was used to assess the dynamic rheological performance of the modified binders and basic bitumen (Blank). As the temperature rose, all samples showed a gradual decrease, signifying the change from a semi-solid to a more fluid, viscous state^33,34^. The binder stiffness was significantly increased by the addition of chemically modified polystyrene (PS-g-AM) at all tested temperatures. The curves changed higher when the PS-g-AM level rose from 3wt% to 10wt%, indicating a distinct dose-response relationship. Out of all the chemically treated samples, the 10wt% PS-g-AM modification produced the highest modulus^35^.

This implies that stronger intermolecular interactions, most likely hydrogen bonding between the polymer chains and the asphaltene micelles inside the bitumen, are facilitated by the polar functional groups introduced by the grafting of acrylic acid followed by amidation. In comparison to the base bitumen, this produces a more stable, reinforced microstructural network that is more resistant to thermal softening.

The performance difference between the chemically modified PS-g-AM and the untreated waste PS with an equivalent loading of 7wt% is a noteworthy finding in this research. Compared to the 7wt% PS-g-AM, the untreated waste PS showed a significantly higher complex modulus. This phenomenon can be explained by the different mechanisms of reinforcement; the untreated waste polystyrene remains as a discrete, high-modulus solid phase that physically obstructs the flow of the binder, resulting in a “brute-force” (Physical Reinforcement). Conversely, PS-g-AM improves the polarity and compatibility of the polystyrene through chemical interaction via grafting with aliphatic chains hydrocarbon and amidation (Chemical Integration). This leads to internal plasticization and better dispersion within the maltene phase that may slightly reduce the absolute stiffness^35,36^.

#### Phase angle (δ)

**Fig. 7:**
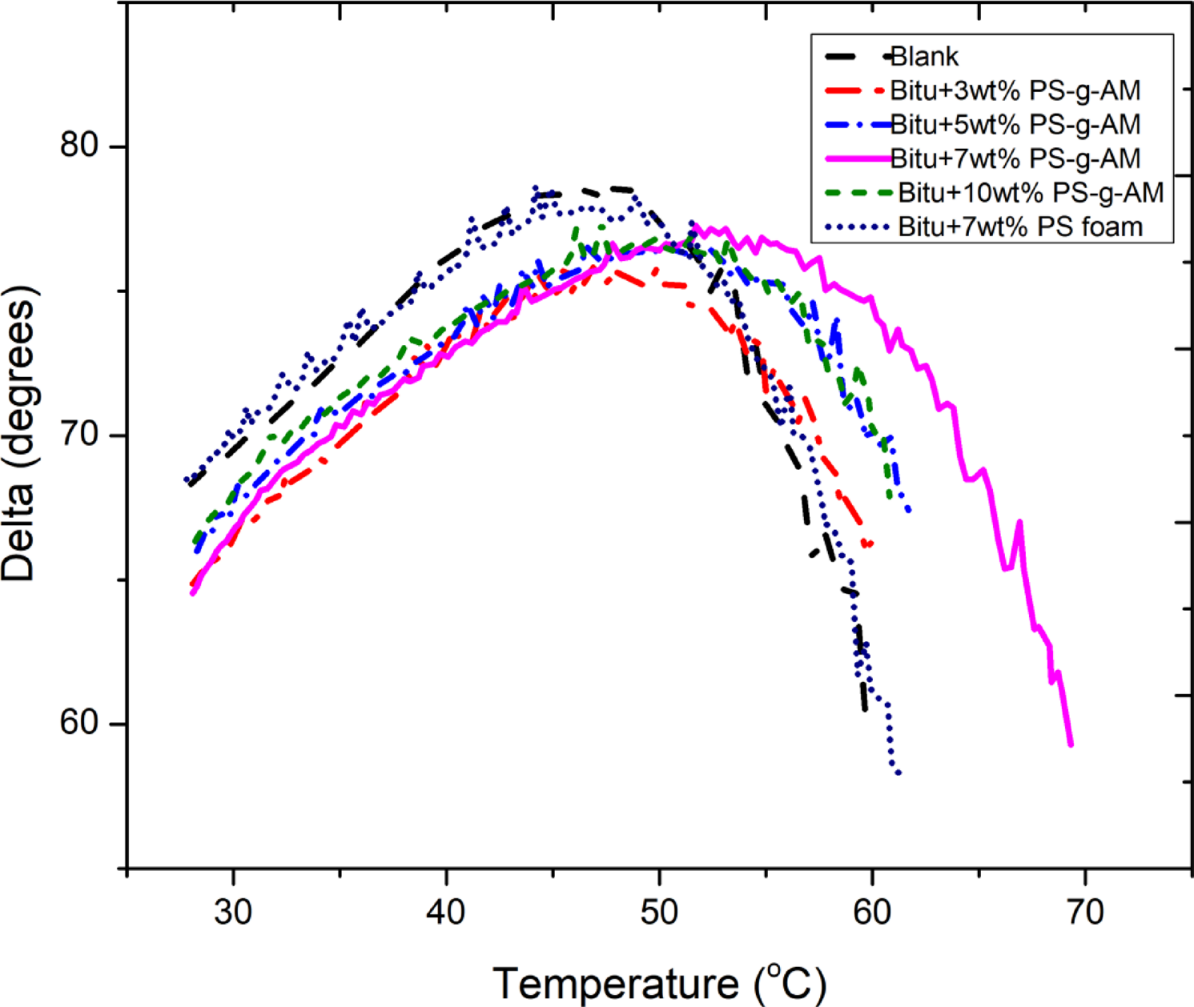
Phase angle (δ) behavior of blank, and bitumen modified by PS, and PS-g-AM.

As shown in Fig. 7, the phase angle data throughout a temperature range of 25 °C to 70 °C offer crucial information on the molecular mobility and structural integrity of the modified binders^28^. As the temperature increases from 30 °C to about 50 °C, the base bitumen (Blank) and modified samples first exhibit an increase. This pattern reflects the bitumen matrix’s anticipated thermal softening, which causes the substance to become more viscous. However, the changed samples show a clear “plateau” or decreasing trend at higher temperatures (over 50 °C), a feature typical of the development of a stable polymer network. At high temperatures, the Bitu + 7wt% PS-g-AM shows the best improvement in elastic response. This is essential to avoid long-term pavement deformation. In comparison to the treated 7wt% PS-g-AM over a large portion of the range, the phase angle curve for an equivalent loading of 7wt% from untreated waste PS is closer to the Blank bitumen at lower temperatures. This supports the idea that untreated PS serves mainly as a physical filler, giving the binder rigidity without necessarily improving its underlying elastic network. On the other hand, the PS-g-AM samples’ smaller phase angle indicates a high chemical compatibility that enables the polymer to actually alter the bitumen’s viscoelastic properties rather than merely raising its bulk viscosity.

#### Rutting factor parameter (G*/sin δ)

**Fig. 8:**
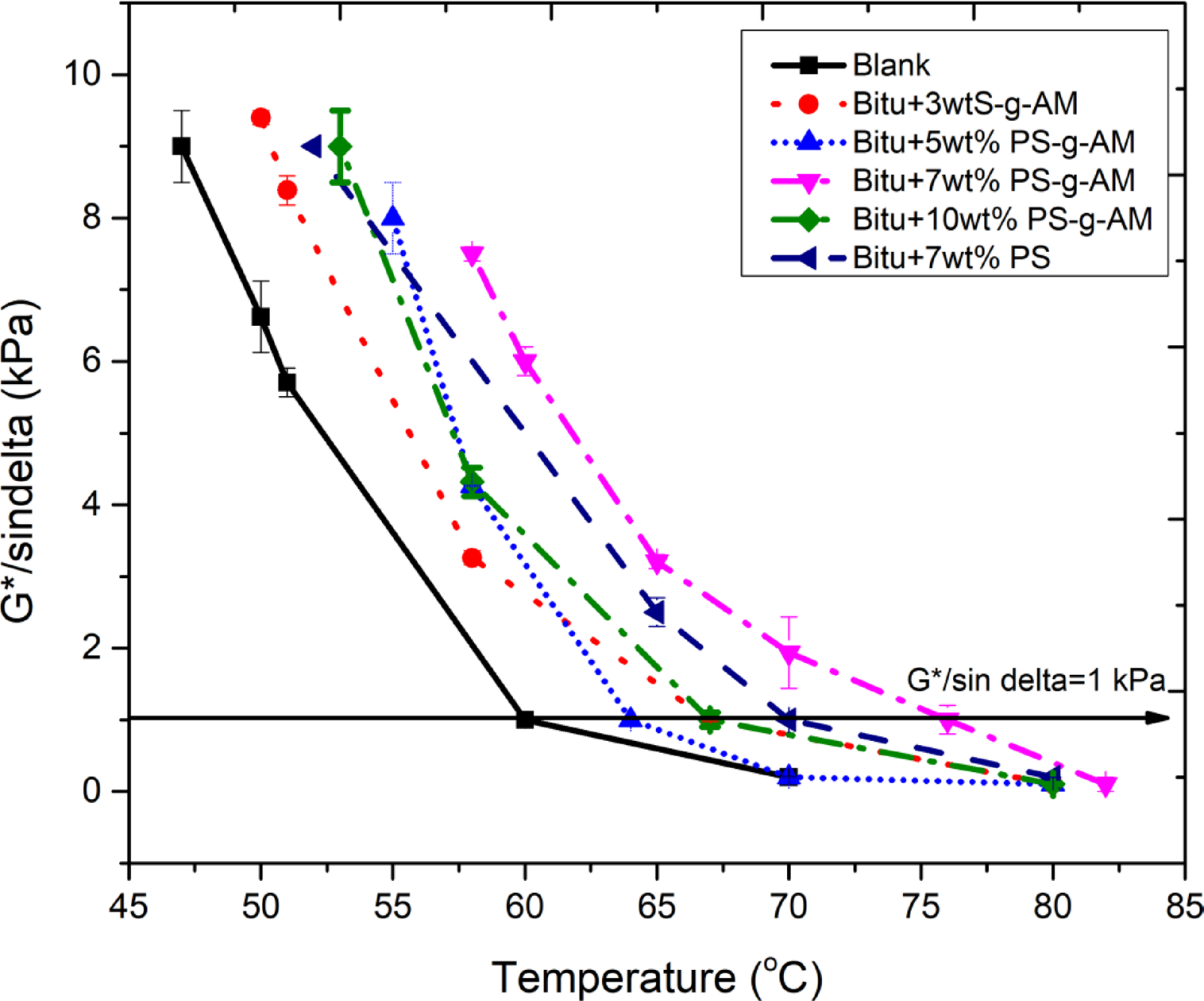
Rutting factor for unaged Blank, and bitumen modified by PS, and PS-g-AM.

**Fig. 9:**
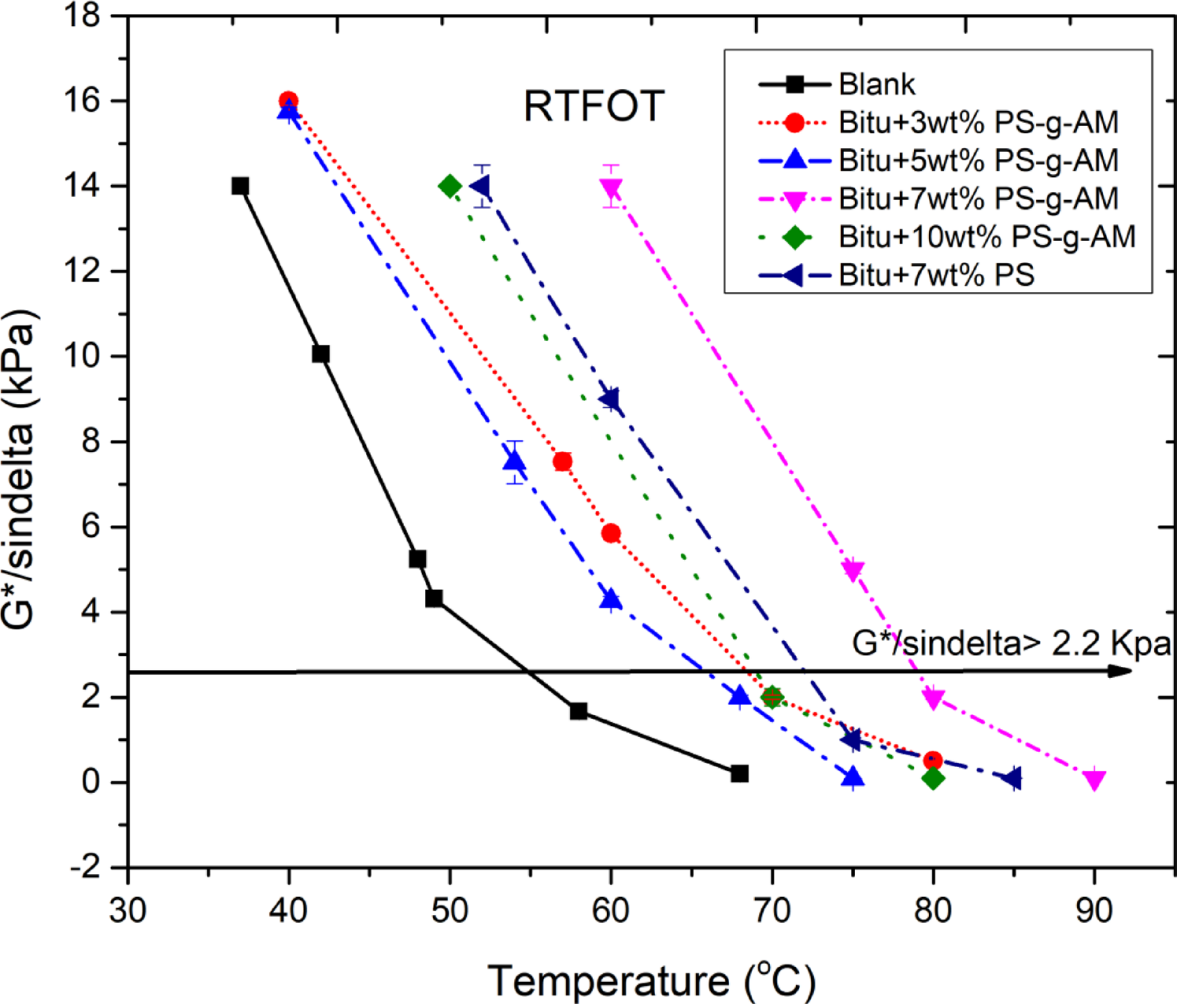
Rutting factor for aged blank, and bitumen modified by PS, and PS-g-AM.

The rutting parameter (G*/sin δ) reflects the combined effects of reduced phase angle (δ) and increased stiffness (G*)^37–39^. The blank bitumen has the lowest G*/sin δ values, as shown in Fig. 8. It reaches the limiting value of G∗/sin/δ = 1.0 (KPa) at about 58 °C, and satisfies the criterion of G∗/sinδ ≥ 2.2 kPa after RTFOT as shown in Fig. 9, which corresponds to a PG 58 high-temperature grade. This comparatively low threshold temperature validates the unmodified binder’s weak resistance to permanent deformation. On the other hand, All PS-g-AM-modified bitumen, exhibits noticeably greater rutting parameter values, and performance gets better as the polymer content in the bitumen increases. Up to around 64 °C, the (Bitu + 3 wt% PS-g-AM) retains G∗/sinδ ≥ 1.0 kPa, and meets the aging criterion up to 64 °C which is equivalent to a PG 64 grade. The critical temperature rises to about 70 °C when the polymer content is increased to 5 wt%, meeting PG 70 criteria before and after RTFOT for (Bitu+5wt%PS-g-AM). It is noticed that, the (Bitu + 7 wt% of PS-g-AM) shows the strongest resilience to temperature-induced softening, retaining the Superpave threshold up to roughly 76–78 °C, which is commensurate with a PG 76 classification. Conversely, the critical temperature is not considerably raised by 10 wt% PS-g-AM binder compared to the 7 wt% formulation, suggesting diminishing returns at larger polymer dosages. This might be caused by an overabundance of polymers, which would increase phase rigidity or decrease dispersion efficiency. Also, the untreated waste polystyrene reaches the kPa failure threshold at a lower temperature than the 7 wt% PS-g-AM sample, demonstrating that untreated polymer is less effective at extending the high-temperature service range of the binder.

### Multi-scale correlation between microscopic mechanisms and macroscopic performance

This section shows how the chemical modification of waste polystyrene (PS) by grafting and amidation profoundly changes its interaction with bitumen from a simple physical blend to a structured, high-performance binder. The inclusion of polar amine and amide groups, as shown by FTIR and 1 H NMR, allows for the creation of a reversible hydrogen-bonded network with the bitumen’s asphaltene portion. This microscopic connection is visibly validated by AFM, which shows a continuous, nanoscale surface rough (20 nm) that inhibits phase separation and serves as a structural scaffold. This results in a significant improvement in macroscopic characteristics, with elastic recovery increasing from < 25% to 55%, phase angle decreasing, and rutting factor (G*/sinδ) rising to > 2 kPa at 70 °C before aging. Finally, these findings demonstrate that the nanoscale hydrogen-bond network efficiently transfers viscous flow into elastic energy, resulting in superior resistance to permanent deformation.

## Conclusions

1. FT-IR and H^1^NMR analyses confirmed the successful grafting of acrylamide onto the polystyrene backbone, introducing polar amide functional groups that enhance chemical affinity and interaction with bitumen components.
2. Penetration index, elastic recovery and viscosity measurements results confirmed that chemical grafting effectively reduces temperature susceptibility and introduces a strong elastic component with improved workability in contrast to unmodified polystyrene waste, which primarily acts as a rigid stiffening agent.
3. Storage stability evaluation in accordance with ASTM criteria showed negligible differences in softening point and penetration between the upper and lower sections of PS-g-AM -modified binders, confirming excellent resistance to phase separation even at high modifier contents.
4. PS-g-AM improves elastic behavior and resistance to deformation by dramatically increasing complex modulus and decreasing phase angle, and consequently increasing the factor G*/sin delta which represents the rutting resistance as demonstrated by dynamic mechanical analyzer.
5. According to differential scanning calorimetry, PS-g-AM increases bitumen’s thermal stability by shifting thermal transitions to higher temperatures and reducing thermal susceptibility compared to the unmodified binder.
6. The results elucidate that waste polystyrene can be an effective asphalt modifier via chemical grafting with acrylamide, which is a promising pathway to upcycle plastic waste into value-added pavement materials.

## Data Availability

“The authors declare that the data supporting the findings of this study are available within the paper. Should any raw data files be needed in another format, they are available from the corresponding author upon reasonable request. All data that support the findings of this study are included within the article.”
